# Caregivers’ knowledge, attitudes, and practices regarding secondary prevention of stroke in patients with ischemic stroke

**DOI:** 10.1038/s41598-026-51436-8

**Published:** 2026-05-01

**Authors:** Ben Wei, Duozi Wang, Chengqi Yue, Wei Kuang, Qian Yang, Qian Liu, Hengli Peng, Kan Jiang, Jie Huang

**Affiliations:** 1 Department of Neurology, Dujiangyan Shoujia Hospital, Chengdu, 611830 Sichuan China; 2 Department of Neurology, Sichuan Provincial People’s Hospital, University of Electronic Science and Technology of China, Chengdu, 610072 Sichuan China; 3 Department of Neurology, Chinese People’s Liberation Army Western Theater Command General Hospital, Chengdu, 610083 Sichuan China; 4 Department of Neurology, Anyue County People’s Hospital, Anyue, 642350 Sichuan China; 5 Department of Neurology, The first affiliated Hospital of Chengdu Medical College, Chengdu, 610500 Sichuan China; 6 Department of Neurology, Mianyang Third People’s Hospital, Mianyang, 621000 Sichuan China

**Keywords:** Stroke, Caregivers, Secondary Prevention, Health Knowledge, Attitudes, Practice, Cross-Sectional Studies, Diseases, Health care, Medical research, Neurology, Neuroscience

## Abstract

This study aimed to investigate caregivers’ knowledge, attitudes, and practices (KAP) regarding secondary stroke prevention in individuals with ischemic stroke. A cross-sectional survey was administered once per participant during the recruitment period from March 26, 2024 to March 27, 2025 across five hospitals in Sichuan Province, using a standardized questionnaire to gather data. Participants’ demographic information was recorded, and KAP scores related to secondary stroke prevention were assessed. A total of 589 valid questionnaires were analyzed. The majority of participants were female (63.0%), lived in urban areas (70.6%), and had at least an associate degree (34.8%). The scores for knowledge, attitude, and practice were 10.85 ± 4.61 (possible range: 0–22), 34.66 ± 4.82 (possible range: 10–50), and 38.51 ± 6.06 (possible range: 10–50), respectively. Correlation analysis revealed significant positive relationships between knowledge and practice (*r* = 0.123, *P* = 0.003) and between attitude and practice (*r* = 0.338, *P* < 0.001). However, the correlation between knowledge and attitude was not significant. Structural equation modeling indicated that knowledge directly affected practice (β = 0.140, *P* = 0.032), and attitude also directly affected practice (β = 0.253, *P* = 0.016). Overall, caregivers reported lower knowledge scores and less favorable attitudes, while practice scores were comparatively higher within the observed range. Both knowledge and attitude had a direct effect on caregiving behaviors.

**Supplementary Information:**

The online version contains supplementary material available at 10.1038/s41598-026-51436-8.

## Introduction

Ischemic stroke accounts for approximately 87% of all stroke cases and remains a leading cause of mortality and long-term disability worldwide^1,2^. Survivors of ischemic stroke are at high risk of recurrence, with annual recurrent stroke rates ranging from 5% to 21%^3^. Recurrent strokes significantly increase the risk of death and disability, placing a substantial burden on patients, families, and healthcare systems^4^. Evidence indicates that structured secondary prevention strategies, encompassing strict control of blood pressure and glucose, lipid management, smoking cessation, and adherence to antithrombotic therapy, can reduce the risk of recurrence by up to 80%^5^. Despite these benefits, the implementation of secondary prevention remains suboptimal, particularly in low-resource settings or regions with marked disparities in healthcare access, such as parts of China^6^.

Caregivers play a pivotal role in post-discharge care by supporting long-term secondary prevention of ischemic stroke, as their awareness of recurrence prevention directly influences both the quality of care and patient adherence to preventive measures^7^. It is estimated that over 80% of stroke survivors rely on caregivers, who provide an average of 35 h of care per week^8^. However, existing studies indicate that many caregivers lack adequate knowledge about stroke prevention, which may adversely affect caregiving behaviors and patient outcomes. For instance, recent findings from Iran and China revealed that although caregivers generally exhibit positive attitudes, they demonstrate only moderate knowledge and inconsistent preventive practices^9,10^.

The Knowledge, Attitude, and Practices (KAP) survey serves as a diagnostic tool to assess individuals’ cognitive, affective, and behavioral dimensions concerning specific health issues^11^. Widely applied in public health research, the KAP framework is based on the premise that increased knowledge promotes positive attitudes, which in turn lead to health-promoting behaviors^12^. This model has been used across various disease areas to assess awareness, identify misconceptions, and inform the design of targeted educational interventions^13^. In the context of ischemic stroke, KAP studies have primarily focused on patients, evaluating their awareness of risk factors and engagement in preventive behaviors^14^. In contrast, caregiver-related studies have mainly addressed general caregiving during rehabilitation, with limited emphasis on their role in secondary prevention^15^.

To address this gap, this study aimed to assess caregivers’ levels of knowledge, attitudes, and practices regarding secondary prevention after ischemic stroke and to examine the relationships among these KAP constructs using correlation analysis and structural equation modeling in a multicenter sample from Sichuan Province, China.

## Methods

### Study design and participants

The present cross-sectional study was carried out between March 26, 2024 and March 27, 2025 at five hospitals located in Sichuan Province: Sichuan Provincial People’s Hospital, Dujiangyan Shoujia Hospital, the General Hospital of Western Theater Command, Mianyang Third People’s Hospital, and Anyue County People’s Hospital. During this period, eligible caregivers were recruited at each participating hospital, and the questionnaire was completed at a single time point for each participant (at discharge or shortly after discharge), with no follow-up assessments. The study population consisted of caregivers responsible for patients diagnosed with ischemic stroke. Inclusion criteria: (1) Family members or guardians who live with the patient or visit daily; (2) Provided an average of ≥ 4 h of care per day over the past 3 months. Exclusion criteria: (1) Professional caregivers (whose behavior may be influenced by occupational training); (2) Temporary caregivers (total cumulative caregiving duration < 3 months); (3) Individuals with severe cognitive impairment or mental illness. This study was approved by the Medical Ethics Committee of Shoujia Hospital, Dujiangyan (Clinical trial review [2023] No. 001) on November 20, 2023. Prior to participation, written informed consent was obtained from all individuals involved in the study, in accordance with established ethical standards.

## Questionnaire introduction

The questionnaire was developed with reference to the Chinese Guidelines for the Prevention and Treatment of Stroke (2021 Edition) and the Chinese Guidelines for the Secondary Prevention of Ischemic Stroke and Transient Ischemic Attack (2022 Edition)^16,17^. A preliminary pilot study was conducted with a small sample of participants (*n* = 35) to assess clarity and reliability, yielding a reliability coefficient of 0.879, indicating acceptable internal consistency.

The finalized questionnaire, administered in Chinese, comprised four sections: demographic information, and the three domains of knowledge, attitude, and practice (KAP) (Appendix). The demographic section captured data on both caregivers (including age, sex, place of residence, educational attainment, occupational status, relationship to the patient, and cohabitation status) and patients (including age, sex, marital status, height, weight, residence, education level, per capita monthly household income, type of medical insurance, history of cerebrovascular disease, frequency of stroke or transient ischemic attack, comorbidities, smoking and alcohol consumption status, dietary patterns, and the time interval between symptom onset and seeking medical care).

The knowledge section included nine items assessing familiarity with key aspects of stroke recurrence prevention. Responses were scored as follows: “Very familiar” = 2 points, “Somewhat familiar” = 1 point, and “Unfamiliar” = 0 points. A three-option familiarity scale was used to reduce respondent burden and facilitate comprehension in caregivers with heterogeneous educational backgrounds, as the knowledge items aimed to capture perceived familiarity rather than agreement. The final item in this section permitted multiple responses, each scored 1 point, with a maximum of six points. The total possible score for the knowledge dimension ranged from 0 to 22. The attitude section comprised ten items measured on a 5-point Likert scale ranging from “Strongly agree” (5 points) to “Strongly disagree” (1 point), with a total score range of 10 to 50. Similarly, the practice section included ten items rated on a 5-point Likert scale, from “Always/Very consistent” (5 points) to “Never/Very inconsistent” (1 point), also yielding a total possible score between 10 and 50. Higher scores indicate greater familiarity (knowledge), more positive attitudes, and more consistent practices regarding secondary stroke prevention.

## Questionnaire distribution and quality control

The survey was conducted in four cities and counties in Sichuan Province—Chengdu, Mianyang, Dujiangyan, and Anyue—with Dujiangyan and Anyue selected as representative sites for primary healthcare settings. Recruitment was conducted in the neurology inpatient wards and stroke follow-up clinics of the participating hospitals. Trained investigators (attending neurologists or neurology-trained nurses) screened potential participants using medical records and approached eligible primary family caregivers at follow-up visits, during bedside caregiving, or at discharge education sessions. Caregivers who agreed to participate received a paper invitation including a written informed consent form and a unique QR code linking to the standardized electronic questionnaire platform, which they typically completed on site using their own mobile device, with operational assistance available when needed. The number of caregivers who were approached but declined participation was not systematically recorded. At follow-up visits, during bedside caregiving, or at discharge education sessions, trained investigators introduced the study face-to-face and provided eligible caregivers with a paper invitation that included a written informed consent form and a unique QR code linking to the standardized electronic questionnaire platform; caregivers accessed the survey only after signing the consent form.

To ensure methodological rigor and maintain data integrity, a project management committee composed of senior representatives from the five participating hospitals held monthly virtual meetings to supervise data validation and quality control procedures. Each study site designated personnel responsible for distributing questionnaires and verifying responses, which enhanced the accuracy and completeness of the dataset. The clinical staff involved included neurologists at the attending level or higher, along with neurology-trained nurses experienced in stroke management. Caregivers completed the questionnaire with guidance from trained healthcare professionals. For older participants or those with limited literacy, questions were read aloud to aid comprehension, and all responses were reviewed for accuracy before final submission.

### Statistical analysis

Data analysis was performed using SPSS version 27.0 (IBM Corp., Armonk, NY, USA) and Amos version 26.0 (IBM Corp., Armonk, NY, USA). Continuous variables are presented as means and standard deviations (SD), while categorical variables are expressed as frequencies and percentages (n, %). The distribution of continuous data was assessed for normality. For comparisons between two groups, the independent sample t-test was used when data followed a normal distribution, whereas the Wilcoxon Mann–Whitney test was applied for non-normally distributed data. When comparing three or more groups, one-way analysis of variance (ANOVA) was used for normally distributed variables with homogeneity of variance, while the Kruskal–Wallis test was employed for variables that were not normally distributed. Correlation analyses were conducted to explore associations among knowledge, attitude, and practice (KAP) scores. Depending on the distribution of the data, Pearson or Spearman correlation coefficients were used. Structural equation modeling (SEM) was conducted to further examine the directional relationships among the three latent constructs—knowledge, attitude, and practice. The hypothesized paths included: (1) a direct effect of knowledge on attitude; (2) a direct effect of attitude on practice; and (3) both direct and indirect effects of knowledge on practice. Knowledge, Attitude, and Practice were specified as latent variables measured by K1–K9, A1–A10, and P1–P10, respectively. Indirect effects were evaluated using bootstrapping and are reported with confidence intervals. A limited number of correlated residuals were allowed and MI-guided adjustments (MI > 20) were applied. Model fit was evaluated using multiple indices, including the root mean square error of approximation (RMSEA), incremental fit index (IFI), Tucker–Lewis index (TLI), and comparative fit index (CFI)^18^. All statistical tests were two-sided, and a P-value < 0.05 was considered indicative of statistical significance.

## Results

This study initially collected 609 questionnaires. However, certain responses were excluded: 1) 3 cases from respondents under 18 years old; 2) 13 cases with clearly implausible patient height/weight entries (suggesting data entry errors or misreporting, e.g., two-digit heights or unrealistically high body weights); and 3) 4 cases with logical inconsistencies in multiple-choice question 11. As a result, 589 valid responses remained, representing 96.72% of the collected questionnaires. Internal consistency was examined for each domain separately, and the corresponding indices are reported in Supplementary Table 1. Assessment of the instrument’s distribution revealed no significant floor or ceiling effects across the three dimensions. Specifically, the ceiling effect for the ‘Practice’ dimension was the highest at 10.5%, followed by ‘Knowledge’ (2.1%) and ‘Attitude’ (0.6%), while all floor effects were negligible (≤ 0.3%) (Supplementary Table 2). The CFA showed acceptable model fit (CMIN/DF = 3.239, RMSEA = 0.060, IFI = 0.902, TLI = 0.890, and CFI = 0.902).

### Demographic information on participants

The final sample included 589 caregivers of ischemic stroke patients, predominantly female (63.0%) and urban residents (70.6%), with 34.8% holding associate or bachelor’s degrees or higher. Most caregivers were responsible for elderly patients (46.0% aged 71 years or older) who had hypertension (61.5%).

The caregivers’ knowledge, attitude, and practice scores were 10.85 ± 4.61, 34.66 ± 4.82, and 38.51 ± 6.06, respectively. Caregivers’ knowledge and attitude scores differed by several caregiver characteristics. Higher caregiver education was associated with higher knowledge and attitude scores (both *P* < 0.001), and caregivers living in urban areas reported higher knowledge and attitude scores than those in rural areas (*P* = 0.004 and *P* = 0.041, respectively). Knowledge scores also differed by occupation type (*P* = 0.020), and attitude scores differed by the caregiver–patient relationship (*P* = 0.003). Practice scores were higher among caregivers living with the patient than those not cohabiting (*P* = 0.014). In addition, caregiver knowledge scores varied across several patient characteristics, including patient education level (*P* = 0.016), per-capita monthly household income (*P* = 0.011), medical insurance type (*P* = 0.004), history of cerebrovascular events (*P* < 0.001), and number of stroke/TIA occurrences (*P* < 0.001) (Table 1).

**Table 1:** Demographic characteristics and KAP scores.

	*n* (%)	Knowledge		Attitude		Practice	
		mean ± SD	*P*	mean ± SD	*P*	mean ± SD	*P*
Total	589	10.85 ± 4.61		34.66 ± 4.82		38.51 ± 6.06	
Caregiver							
Age	50.60 ± 14.35						
Gender			0.718		0.250		0.094
Male	218 (37.01)	10.83 ± 4.78		35.02 ± 5.03		37.81 ± 6.68	
Female	371 (62.99)	10.86 ± 4.52		34.46 ± 4.69		38.93 ± 5.64	
Residence			0.004		0.041		0.056
Rural	118 (20.03)	10.28 ± 5.25		33.68 ± 4.38		37.03 ± 6.51	
Urban	416 (70.63)	11.18 ± 4.35		34.96 ± 4.87		38.91 ± 5.68	
Suburban	55 (9.34)	9.58 ± 4.86		34.51 ± 5.13		38.73 ± 7.37	
Education			< 0.001		< 0.001		0.798
Primary school or below	105 (17.83)	10.15 ± 5.81		32.84 ± 4.2		38.84 ± 6.44	
Junior high school	151 (25.64)	9.23 ± 4.13		33.74 ± 4.11		38.19 ± 5.85	
High school/Vocational school	128 (21.73)	10.89 ± 3.99		34.98 ± 5.05		38.91 ± 6.21	
Associate degree/Bachelor’s degree or above	205 (34.8)	12.38 ± 4.13		36.08 ± 5.03		38.35 ± 5.95	
Occupation type			0.020		0.171		0.605
Full-time/Part-time/Freelancer	242 (41.09)	11.46 ± 4.64		35.11 ± 5.06		38.19 ± 5.97	
Retired	164 (27.84)	10.26 ± 4.47		34.05 ± 4.45		39.03 ± 6	
Other	183 (31.07)	10.56 ± 4.62		34.62 ± 4.78		38.48 ± 6.24	
Relationship to the Patient			0.064		0.003		0.642
Spouse of the patient	227 (38.54)	10.46 ± 4.44		33.75 ± 4.32		38.94 ± 5.8	
Child of the patient	290 (49.24)	11.21 ± 4.65		35.27 ± 4.96		38.31 ± 6.27	
Other	72 (12.22)	10.6 ± 4.92		35.11 ± 5.34		38 ± 6.05	
Living with the Patient			0.647		0.224		0.014
Yes	396 (67.23)	10.81 ± 4.51		34.44 ± 4.67		38.97 ± 5.83	
No	193 (32.77)	10.94 ± 4.82		35.13 ± 5.1		37.58 ± 6.43	
Patient							
Gender			0.828		0.565		0.502
Male	381 (64.69)	10.87 ± 4.69		34.58 ± 4.7		38.7 ± 6.12	
Female	208 (35.31)	10.82 ± 4.47		34.82 ± 5.05		38.17 ± 5.95	
Age			0.153		0.962		0.918
18–30	6 (1.02)	11.83 ± 4.92		35.33 ± 4.68		36.83 ± 8.06	
31–50	50 (8.49)	10.18 ± 5.03		34.58 ± 4.18		38.62 ± 6.03	
51–70	262 (44.48)	10.6 ± 4.4		34.73 ± 4.9		38.65 ± 6.09	
≥ 71	271 (46.01)	11.19 ± 4.71		34.6 ± 4.88		38.41 ± 6.03	
Marital Status			0.267		0.281		0.674
Married	489 (83.02)	10.79 ± 4.61		34.54 ± 4.74		38.67 ± 5.98	
Unmarried	100 (16.98)	11.15 ± 4.6		35.27 ± 5.17		37.76 ± 6.44	
BMI			0.636		0.087		0.848
Underweight	25 (4.24)	11.24 ± 5.14		32.96 ± 5.5		38.56 ± 5.84	
Normal	346 (58.74)	10.9 ± 4.52		34.89 ± 4.99		38.43 ± 5.9	
Overweight or obese	218 (37.01)	10.72 ± 4.71		34.5 ± 4.43		38.65 ± 6.37	
Residence in the Past Year			0.185		0.082		0.045
Rural	153 (25.98)	10.75 ± 5.14		33.97 ± 4.65		37.44 ± 6.09	
Urban	370 (62.82)	11.03 ± 4.33		34.92 ± 4.93		39.01 ± 5.82	
Suburban	66 (11.21)	10.05 ± 4.81		34.85 ± 4.51		38.2 ± 7.03	
Education			0.016		0.364		0.586
Primary school or below	233 (39.56)	10.42 ± 5.24		34.36 ± 4.79		37.98 ± 6.58	
Junior high school	185 (31.41)	10.7 ± 4.05		34.64 ± 4.96		38.77 ± 5.75	
High school/Vocational school	91 (15.45)	11.19 ± 4.25		34.96 ± 4.69		39.05 ± 5.8	
Associate degree/Bachelor’s degree or above	80 (13.58)	12.04 ± 4.08		35.28 ± 4.75		38.86 ± 5.45	
Monthly income per capita			0.011		0.084		0.374
<2000	148 (25.13)	10.16 ± 5.35		34.12 ± 4.81		38.37 ± 6.52	
2000–5000	275 (46.69)	10.71 ± 4.13		34.67 ± 4.65		38.91 ± 5.99	
5000–10,000	129 (21.9)	11.54 ± 4.68		34.66 ± 4.79		37.78 ± 5.72	
> 10,000	37 (6.28)	12.19 ± 4.14		36.78 ± 5.74		38.76 ± 5.83	
Medical insurance type			0.004		0.088		0.078
No medical insurance	30 (5.09)	10.6 ± 5.73		35 ± 6.3		37.17 ± 7.11	
Social insurance only	473 (80.31)	10.57 ± 4.34		34.43 ± 4.57		38.4 ± 6.08	
Social insurance and additional commercial insurance	86 (14.6)	12.47 ± 5.3		35.81 ± 5.44		39.62 ± 5.46	
History of Cerebrovascular Events			< 0.001		0.056		0.528
Yes	174 (29.54)	12.14 ± 4.57		33.98 ± 4.5		38.76 ± 5.55	
No	415 (70.46)	10.31 ± 4.52		34.95 ± 4.93		38.41 ± 6.27	
Number of Stroke or TIA Occurrences			< 0.001		0.155		0.993
First	417 (70.8)	10.45 ± 4.61		34.97 ± 4.92		38.47 ± 6.22	
Second	120 (20.37)	11.15 ± 4.56		34.15 ± 4.66		38.58 ± 5.76	
Third	29 (4.92)	13.76 ± 4.66		33.34 ± 4.2		38.86 ± 5.74	
More than three	23 (3.9)	12.83 ± 2.81		33.52 ± 4		38.61 ± 5.48	
Hypertension			0.650		0.056		0.836
Yes	362 (61.46)	10.9 ± 4.53		34.35 ± 4.74		38.43 ± 6.04	
No	227 (38.54)	10.77 ± 4.75		35.17 ± 4.92		38.65 ± 6.11	
Hyperlipidemia			0.005		0.107		0.487
Yes	137 (23.26)	11.88 ± 4.26		34.06 ± 4.5		38.07 ± 4.89	
No	452 (76.74)	10.54 ± 4.67		34.85 ± 4.9		38.65 ± 6.37	
Diabetes Mellitus			0.430		0.014		0.101
Yes	211 (35.82)	11 ± 4.33		33.91 ± 4.15		39.05 ± 6.02	
No	378 (64.18)	10.76 ± 4.76		35.08 ± 5.11		38.22 ± 6.08	
Heart Disease			0.499		0.717		0.034
Yes	91 (15.45)	11.14 ± 5.15		34.82 ± 4.97		39.7 ± 7.01	
No	498 (84.55)	10.8 ± 4.51		34.63 ± 4.8		38.3 ± 5.86	
Smoking Habits			0.836		0.441		0.080
Yes	192 (32.6)	10.83 ± 4.75		34.56 ± 5.05		39.18 ± 6.1	
No	397 (67.4)	10.86 ± 4.55		34.72 ± 4.71		38.19 ± 6.03	
Alcohol Consumption			0.285		0.102		0.404
Yes	173 (29.37)	11.06 ± 4.54		35.19 ± 4.75		38.92 ± 5.82	
No	416 (70.63)	10.76 ± 4.64		34.44 ± 4.84		38.35 ± 6.16	
Preference for High-Salt/High-Fat Diet			0.815		0.056		0.983
Yes	287 (48.73)	10.83 ± 4.57		34.3 ± 4.72		38.51 ± 6.15	
No	302 (51.27)	10.87 ± 4.66		35.01 ± 4.9		38.52 ± 5.99	

## Knowledge, attitude, and practice

The distribution of knowledge dimensions showed that the three questions with the highest number of participants choosing the ‘Unfamiliar’ option were ‘Stroke refers to an interruption in blood supply to the brain, resulting in localized cerebral ischemia, hypoxia, and necrosis. It is a common cerebrovascular disease, also known as “ischemic stroke.“’ (K1) with 36.33%, ‘Long-term medication is needed to prevent stroke recurrence, along with control of underlying conditions; cerebrovascular interventions or surgeries may be necessary in some cases.’ (K4) with 34.8%, and ‘Medications to prevent stroke recurrence are more effective when started as early as possible after onset.’ (K3) with 28.86%. When it comes to risk factors for cerebrovascular disease, hypertension (90.15%) is the most widely known, followed by hyperlipidemia (70.97%) and diabetes (70.29%) (Table 2).

**Table 2:** Distribution of knowledge dimension responses.

Knowledge	*N* (%)		
	Very familiar	Somewhat familiar	Unfamiliar
1. Stroke refers to an interruption in blood supply to the brain, resulting in localized cerebral ischemia, hypoxia, and necrosis. It is a common cerebrovascular disease, also known as “ischemic stroke.”	31 (5.26)	344 (58.4)	214 (36.33)
2. Recurrent stroke significantly increases the risk of death and disability.	46 (7.81)	375 (63.67)	168 (28.52)
3. Medications to prevent stroke recurrence are more effective when started as early as possible after onset.	68 (11.54)	351 (59.59)	170 (28.86)
4. Long-term medication is needed to prevent stroke recurrence, along with control of underlying conditions; cerebrovascular interventions or surgeries may be necessary in some cases.	55 (9.34)	329 (55.86)	205 (34.8)
5. Psychological counseling should be provided to stroke patients to help alleviate anxiety.	74 (12.56)	372 (63.16)	143 (24.28)
6. Stroke patients with hemiplegia have weaker immune function and therefore require enhanced nutrition and infection prevention.	65 (11.04)	388 (65.87)	136 (23.09)
7. Stroke requires long-term healthcare and places a high demand on caregiving.	71 (12.05)	365 (61.97)	153 (25.98)
8. Stroke patients should maintain personal hygiene, a healthy diet, regular physical activity, and good sleep habits.	116 (19.69)	393 (66.72)	80 (13.58)
9. Risk factors for cerebrovascular disease include:			
Hyperlipidemia	418 (70.97)		
Hypertension	531 (90.15)		
Diabetes	414 (70.29)		
Smoking	340 (57.72)		
High-salt diet	403 (68.42)		
Obesity	315 (53.48)		

Responses to the attitude dimension showed that 6.62% strongly agreed and 26.32% agreed that their responsibility is only to provide daily care and responsibility is only to provide daily care them (A3), 4.07% strongly agreed and 23.94% agreed that preventing stroke recurrence is not necessary for recovery and that directly proceeding with acupuncture or other rehabilitation treatments is more effective (A7), and 2.38% strongly agreed and 31.92% agreed that they feel that taking care of a stroke patient’s daily needs is boring and physically exhausting (A9) (Table 3).

**Table 3:** Distribution of attitude dimension response.

Attitude, *n* (%)	Strongly disagree	Disagree	Neutral	Agree	Strongly agree
1. You believe that preventing stroke recurrence in patients is very important.	5(0.85)	2(0.34)	76(12.9)	336(57.05)	170(28.86)
2. You are willing to obtain information related to preventing stroke recurrence through various sources such as the internet, television, or books.	5(0.85)	17(2.89)	95(16.13)	389(66.04)	83(14.09)
3. You think that the recovery of stroke patients is largely unrelated to you; your role is simply to look after them.	93(15.79)	219(37.18)	83(14.09)	155(26.32)	39(6.62)
4. You believe that the medications or treatments required for preventing stroke recurrence consume too much of your energy, making you feel irritated or anxious.	52(8.83)	213(36.16)	147(24.96)	166(28.18)	11(1.87)
5. You believe that improving the patient’s quality of life is crucial for preventing stroke recurrence.	4(0.68)	10(1.7)	78(13.24)	395(67.06)	102(17.32)
6. You think that preventing stroke recurrence requires not only attention to physical health but also psychological counseling.	4(0.68)	8(1.36)	75(12.73)	374(63.5)	128(21.73)
7. You believe that preventing stroke recurrence is not necessary for recovery and that directly proceeding with acupuncture or other rehabilitation treatments is more effective.	59(10.02)	230(39.05)	135(22.92)	141(23.94)	24(4.07)
8. You think that quitting smoking and drinking and changing the patient’s previous lifestyle habits are difficult for both the patient and yourself.	48(8.15)	181(30.73)	104(17.66)	214(36.33)	42(7.13)
9. You feel that taking care of a stroke patient’s daily needs is boring and physically exhausting.	47(7.98)	205(34.8)	135(22.92)	188(31.92)	14(2.38)
10. You believe the patient’s confidence in recovery affects your caregiving attitude and enthusiasm.	29(4.92)	114(19.35)	141(23.94)	276(46.86)	29(4.92)

Responses to the practice dimension showed that 20.88% rarely and 7.13% never actively seek knowledge related to secondary stroke prevention and caregiving through various channels (P1), 7.81% rarely and 3.4% never guide the patient to engage in rehabilitation exercises, quit smoking and drinking, and maintain healthy lifestyle habits (P2), 10.53% rarely and 2.89% never provide psychological support and emotional counseling to help the patient manage negative emotions (P3) (Table 4).

**Table 4:** Distribution of practice dimension responses.

Practice, *n* (%)	Always	Often	Sometimes	Rarely	Never
1. You actively seek knowledge related to secondary stroke prevention and caregiving through various channels (e.g., books, internet, communication with doctors).	66 (11.21)	135 (22.92)	223 (37.86)	123 (20.88)	42 (7.13)
2. You guide the patient to engage in rehabilitation exercises, quit smoking and drinking, and maintain healthy lifestyle habits.	125 (21.22)	268 (45.5)	130 (22.07)	46 (7.81)	20 (3.4)
3. You provide psychological support and emotional counseling to help the patient manage negative emotions.	103 (17.49)	245 (41.6)	162 (27.5)	62 (10.53)	17 (2.89)
4. You assist the patient in maintaining personal and environmental hygiene (e.g., helping them change into clean clothes, maintain oral hygiene, and keep their rest area clean).	146 (24.79)	269 (45.67)	137 (23.26)	31 (5.26)	6 (1.02)
5. You provide nutritional support to the patient (e.g., preparing light and healthy meals).	141 (23.94)	270 (45.84)	127 (21.56)	43 (7.3)	8 (1.36)
	**Fully Compliant**	**Compliant**	**Neutral**	**Not Compliant**	**Very Not Compliant**
6. You exchange experiences with other stroke caregivers regarding stroke recurrence prevention.	101 (17.15)	301 (51.1)	147 (24.96)	38 (6.45)	2 (0.34)
7. You accompany the patient to follow-up visits regularly and ensure adherence to medical advice while monitoring their recovery.	186 (31.58)	339 (57.56)	57 (9.68)	5 (0.85)	2 (0.34)
8. You pay attention to the patient’s cardiovascular and cerebrovascular health and support interventions such as endovascular procedures if needed.	145 (24.62)	305 (51.78)	118 (20.03)	16 (2.72)	5 (0.85)
9. You monitor the patient’s communication with doctors and assist in enhancing their disease self-management skills.	170 (28.86)	361 (61.29)	47 (7.98)	9 (1.53)	2 (0.34)
10. You are consciously attentive to the patient’s needs and physical signs, monitoring indicators such as blood pressure and blood glucose, and provide comprehensive care.	214 (36.33)	322 (54.67)	43 (7.3)	7 (1.19)	3 (0.51)

## Correlation analysis

In the correlation analysis, significant positive correlations were found between knowledge and practice (*r* = 0.123, *P* = 0.003) as well as attitude and practice (*r* = 0.338, *P* < 0.001). However, the correlation between knowledge and attitude was not significant (Table 5).

**Table 5:** Correlation analysis.

Knowledge	Knowledge	Attitude	Practice
	1		
Attitude	0.025 (*P* = 0.543)	1	
Practice	0.123 (*P* = 0.003)	0.338 (*P* < 0.001)	1

### SEM analysis

**Fig. 1:**
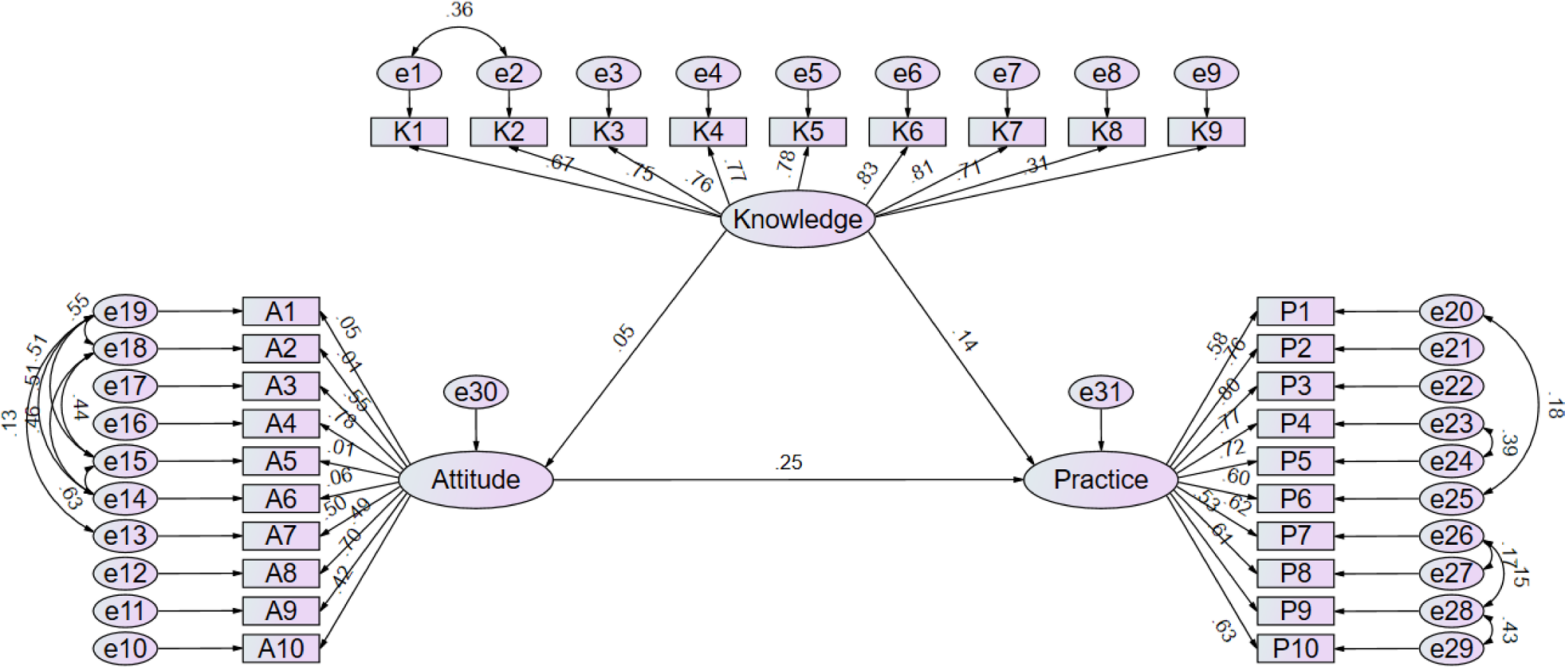
SEM model.

The SEM demonstrated a highly favorable model fit indices (CMIN/DF value: 2.787, RMSEA value: 0.055, IFI value: 0.915, TLI value: 0.904, and CFI value: 0.915) (Supplementary Table 3). The model was specified as a structural measurement model with Knowledge, Attitude, and Practice as latent constructs measured by K1–K9, A1–A10, and P1–P10. Indirect effects were evaluated using bootstrapping, and correlated residuals and MI-guided adjustments (MI > 20). In the structural model, knowledge directly affected practice (β = 0.140, *P* = 0.032), and attitude also directly affected practice (β = 0.253, *P* = 0.016) SEM results show that knowledge directly affected practice (β = 0.140, *P* = 0.032), and attitude also directly affected practice (β = 0.253, *P* = 0.016) (Table 6; Fig. 1).

**Table 6:** SEM results.

Model paths	Standardized Total effects		Standardized direct effects		Standardized indirect effects	
	β (95%CI)	*P*	β (95%CI)	*P*	β (95%CI)	*P*
Knowledge→Attitude	0.048 (-0.083,0.154)	0.526	0.048 (-0.083,0.154)	0.526		
Knowledge→Practice	0.152 (0.032,0.250)	0.023	0.140 (0.015,0.235)	0.032		
Attitude→Practice	0.253 (0.134,0.362)	0.016	0.253 (0.134,0.362)	0.016		
Knowledge→Practice					0.012 (-0.021,0.046)	0.543

## Discussion

Caregivers showed inadequate knowledge, suboptimal attitudes, and generally positive but inconsistent practices toward secondary stroke prevention in individuals with ischemic stroke.

However, because we did not collect objective clinical outcomes (e.g., recurrent stroke events, post-stroke complications, or readmissions) during follow-up, we were unable to quantify how caregivers’ KAP translated into measurable patient outcomes in this study. These findings highlight the critical need for targeted caregiver education and engagement strategies to enhance their understanding and motivation, thereby strengthening adherence to evidence-based secondary prevention measures in clinical practice.

Among the findings of this study, the direct effect of caregivers’ attitudes on preventive practices emerged as particularly consequential. Although knowledge exhibited a weak correlation with behavior, it was the attitudinal dimension that showed a stronger and more consistent relationship with practice. This pattern is consistent with evidence that the translation from cognitive or motivational states to actual behavior is often incomplete (the intention–behavior gap)^19,20^. While these frameworks suggest that cognitive awareness comes before motivation and action, the data presented here indicate a less predictable sequence. Caregivers with limited conceptual understanding of stroke prevention nonetheless maintained a relatively high level of engagement in daily preventive behaviors, suggesting that routine, caregiving expectations, or institutional reinforcement may substitute for formal knowledge in driving practical adherence^21^.

The absence of a significant link between knowledge and attitude further complicates this trajectory. In principle, awareness of disease mechanisms, treatment strategies, and recurrence risks should influence how caregivers perceive their role and responsibilities. Yet the results suggest that factual knowledge alone may not be sufficient to alter underlying beliefs or emotional engagement^22^. This may be particularly true in caregiving contexts shaped by cultural obligation or economic necessity, where decisions are driven less by internalized understanding and more by default expectations or external constraints. Furthermore, the considerable demands of caregiving can lead to caregiver burden and fatigue, which are known barriers to sustained engagement in health-promoting behaviors^23^.

These patterns become clearer when considered alongside the overall distribution of KAP scores. On average, caregivers fell below the threshold for adequate knowledge, especially on items concerning early treatment initiation, long-term pharmacological management, and the clinical definition of ischemic stroke. At the same time, the mean attitude score hovered close to the threshold, with considerable variation among respondents. Several caregivers expressed views suggesting detachment from active prevention, including the belief that their responsibilities end with basic daily care or that recurrence prevention is of secondary importance. Despite these reservations, caregiving practices were relatively robust. Most respondents reported routine involvement in supporting healthy behaviors, assisting with follow-up care, and monitoring patients’ clinical status. What emerges, then, is a profile of caregivers who are behaviorally engaged but conceptually and emotionally underprepared^22^.

Socioeconomic and contextual factors appear to reinforce these patterns. Higher educational attainment was consistently associated with stronger knowledge and more favorable attitudes, suggesting that cognitive and affective readiness for preventive caregiving is closely tied to broader structural resources^24^. Urban residence was also associated with improved performance in the knowledge and attitude domains, likely due to greater access to health information, medical support, and social capital. Practice scores showed fewer differences across demographic categories than knowledge or attitude, suggesting that routine caregiving behaviors may be shaped more by daily caregiving context than by caregivers’ knowledge or attitudes alone. Factors such as cohabitation with the patient, severity of comorbidities, and perceived health risks may play a larger role in shaping daily behavior than formal education or location.

The detailed response patterns in each KAP domain offer further insight into where interventions may be most needed. In the knowledge section, items requiring a biomedical understanding of stroke elicited the highest rates of uncertainty. Familiarity with general risk factors, by contrast, was relatively widespread—possibly due to longstanding public health campaigns that focus on hypertension and diabetes. In our sample, uncertainty was more common for items requiring stroke-specific knowledge, whereas recognition of common risk factors (e.g., hypertension, diabetes, and hyperlipidemia) was relatively high. Similarly, in the attitudinal domain, caregivers were more likely to endorse emotionally or physically disengaged views when questions touched on burden, role responsibility, or perceptions of treatment effectiveness. The proportion of caregivers who viewed caregiving as exhausting or redundant with rehabilitation efforts points to the emotional and psychological dimensions of care that are often overlooked in training and policy^21^.

Caregiving practices, while stronger overall, showed weaknesses in areas requiring higher-order initiative. Many caregivers did not actively seek information or offer psychological support, even as they reliably performed physical caregiving tasks. This suggests that while procedural responsibilities are met, the more proactive aspects of prevention, such as those requiring reflection, planning, or coordination, are inconsistently addressed. Such a pattern is consistent with observations from other chronic disease contexts, where practical routines often outpace deeper engagement with disease management^24^.

The evidence presented here does not support interventions that rely exclusively on increasing factual knowledge. Educational content alone may be insufficient for sustained behavior change, and additional support that targets motivational and self-regulatory barriers may be needed^19,20^. Programs that incorporate peer storytelling, role reinforcement, or caregiver-to-caregiver dialogue may offer more traction than conventional informational materials alone^25,26^. Moreover, initiatives that address role strain and clarify the broader significance of caregiver involvement may help shift attitudes in ways that support long-term behavior change.

Resource disparities must also be addressed directly. Digital platforms, if appropriately adapted for different levels of literacy and internet access, could extend the reach of training materials in urban settings. In rural areas, however, face-to-face communication led by community health workers may remain more effective. Multifactorial interventions combining community-led sessions with follow-up materials have been shown effective in similar contexts^27^, with notable gains in knowledge and practical caregiving ability. Rather than pursuing a single unified strategy, future interventions should differentiate by location, education level, and caregiving context to better align content and delivery mechanisms with caregiver capacity.

At the institutional level, greater attention should be paid to integrating caregiver preparation into stroke discharge protocols. While hospital-based training programs often prioritize patients, caregiver education is frequently informal, rushed, or omitted altogether. Embedding structured caregiver assessments and tailored guidance within clinical workflows could improve both engagement and continuity of care^28^. Educational empowerment models that include reinforcement, telephonic reminders, and post-discharge follow-up have also shown promise in enhancing caregiver knowledge retention and compliance^29^.

This study has several limitations that should be considered when interpreting the findings. First, the cross-sectional design limits our ability to establish causal relationships among knowledge, attitudes, and practices. Second, because the study was conducted in a single province, the results may not accurately reflect caregiver populations in other regions of China, where healthcare resources and cultural contexts may vary. Third, the reliance on self-reported data could introduce response and recall bias, particularly in the evaluation of attitudes and practices. Also, as this study relied on self-reported questionnaire data, the possibility of misclassification bias cannot be excluded. Fourth, the questionnaire underwent pilot testing primarily for clarity and internal consistency, but we did not conduct a formal content validity assessment (e.g., Delphi/CVI). We examined the three-factor structure using CFA, which showed acceptable model fit; however, construct validation was not extended to alternative models or independent samples. In addition, the knowledge score combined eight ordinal “familiarity” items with one multiple-response item into a single total score (0–22) without IRT-based weighting, so the knowledge total should be interpreted cautiously. In addition, we did not capture easily measurable clinical outcomes (such as recurrent stroke, identification of post-stroke complications, or readmission rates); therefore, future prospective studies linking caregiver KAP to longitudinal outcomes are warranted. Additionally, we did not systematically record the number of caregivers who declined participation at the initial invitation stage; therefore, an exact refusal rate could not be calculated, which may introduce selection bias.

In conclusion, caregivers of individuals with ischemic stroke demonstrated relatively limited knowledge, less favorable attitudes, and generally positive preventive practices regarding secondary stroke prevention. Importantly, these findings indicated that improvements in preventive practice should not rely on information provision alone. Given the substantially stronger association observed between attitudes and preventive practices, future interventions should prioritize strategies that enhance caregivers’ motivation, role confidence, and perceived capability to act, alongside addressing practical and contextual barriers. Stroke-prevention knowledge remains a necessary foundation; however, it is unlikely to translate into sustained preventive behaviors without parallel efforts to foster supportive attitudes and enabling environments.

## Supplementary Information

Below is the link to the electronic supplementary material.

Supplementary Material 1

Supplementary Material 2

## Data Availability

The datasets generated and/or analyzed during the current study are not publicly available due to the involvement of human participants and privacy concerns but are available from the corresponding author upon reasonable request.
