# Supplementary material for: Caregivers’ knowledge, attitudes, and practices regarding secondary prevention of stroke in patients with ischemic stroke
Source: Sci Rep. 2026 May 1;16:20209. doi: 10.1038/s41598-026-51436-8 (PMC13323690; doi:10.1038/s41598-026-51436-8)
Supplement: Supplementary file 1 — Supplementary Material 1 [file 41598_2026_51436_MOESM1_ESM.docx]

| **Caregivers’ Knowledge, Attitudes, and Practices Regarding Secondary Prevention of Stroke in Patients with Ischemic Stroke Part 1 Demographic characteristics** |
| --- |
| \| **Caregiver** \| \| --- \| \| **Age** \| \| **Gender** \| \| Male \| \| Female \| \| **Residence** \| \| Rural \| \| Urban \| \| Suburban \| \| **Education** \| \| Primary school or below \| \| Junior high school \| \| High school/Vocational school \| \| Associate degree/Bachelor’s degree or above \| \| **Occupation type** \| \| Full-time/Part-time/Freelancer \| \| Retired \| \| Other \| \| **Relationship to the Patient** \| \| Spouse of the patient \| \| Child of the patient \| \| Other \| \| **Living with the Patient** \| \| Yes \| \| No \| \| **Patient** \| \| **Gender** \| \| Male \| \| Female \| \| **Age** \| \| 18-30 \| \| 31-50 \| \| 51-70 \| \| ≥71 \| \| **Marital Status** \| \| Married \| \| Unmarried \| \| **BMI** \| \| Underweight \| \| Normal \| \| Overweight or obese \| \| **Residence in the Past Year** \| \| Rural \| \| Urban \| \| Suburban \| \| **Education** \| \| Primary school or below \| \| Junior high school \| \| High school/Vocational school \| \| Associate degree/Bachelor’s degree or above \| \| **Monthly income per capita** \| \| ＜2000 \| \| 2000-5000 \| \| 5000-10000 \| \| >10000 \| \| **Medical insurance type** \| \| No medical insurance \| \| Social insurance only \| \| Social insurance and additional commercial insurance \| \| **History of Cerebrovascular Events** \| \| Yes \| \| No \| \| **Number of Stroke or TIA Occurrences** \| \| First \| \| Second \| \| Third \| \| More than three \| \| **Hypertension** \| \| Yes \| \| No \| \| **Hyperlipidemia** \| \| Yes \| \| No \| \| **Diabetes Mellitus** \| \| Yes \| \| No \| \| **Heart Disease** \| \| Yes \| \| No \| \| **Smoking Habits** \| \| Yes \| \| No \| \| **Alcohol Consumption** \| \| Yes \| \| No \| \| **Preference for High-Salt/High-Fat Diet** \| \| Yes \| \| No \|   **Part 2 Knowledge** |

| **1.** **Stroke refers to an interruption in blood supply to the brain, resulting in localized cerebral ischemia, hypoxia, and necrosis. It is a common cerebrovascular disease, also known as "ischemic stroke."** | Very familiar | Somewhat familiar | Unfamiliar |
| --- | --- | --- | --- |
| **2. Recurrent stroke significantly increases the risk of death and disability.** | Very familiar | Somewhat familiar | Unfamiliar |
| **3. Medications to prevent stroke recurrence are more effective when started as early as possible after onset.** | Very familiar | Somewhat familiar | Unfamiliar |
| **4. Long-term medication is needed to prevent stroke recurrence, along with control of underlying conditions; cerebrovascular interventions or surgeries may be necessary in some cases.** | Very familiar | Somewhat familiar | Unfamiliar |
| **5. Psychological counseling should be provided to stroke patients to help alleviate anxiety.** | Very familiar | Somewhat familiar | Unfamiliar |
| **6. Stroke patients with hemiplegia have weaker immune function and therefore require enhanced nutrition and infection prevention.** | Very familiar | Somewhat familiar | Unfamiliar |
| **7. Stroke requires long-term healthcare and places a high demand on caregiving.** | Very familiar | Somewhat familiar | Unfamiliar |
| **8. Stroke patients should maintain personal hygiene, a healthy diet, regular physical activity, and good sleep habits.** | Very familiar | Somewhat familiar | Unfamiliar |
| **9. Risk factors for cerebrovascular disease include:** |  |  |  |
| **Hyperlipidemia** |  |  |  |
| **Hypertension** |  |  |  |
| **Diabetes** |  |  |  |
| **Smoking** |  |  |  |
| **High-salt diet** |  |  |  |
| **Obesity** |  |  |  |

| **Part 3 Attitudes**   \| **1. You believe that preventing stroke recurrence in patients is very important.** \| Strongly agree \| Agree \| Neutral \| Disagree \| Strongly disagree \| \| --- \| --- \| --- \| --- \| --- \| --- \| \| **2.** **You are willing to obtain information related to preventing stroke recurrence through various sources such as the internet, television, or books.** \| Strongly agree \| Agree \| Neutral \| Disagree \| Strongly disagree \| \| **3.** **You think that the recovery of stroke patients is largely unrelated to you; your role is simply to look after them.** \| Strongly agree \| Agree \| Neutral \| Disagree \| Strongly disagree \| \| **4.** **You believe that the medications or treatments required for preventing stroke recurrence consume too much of your energy, making you feel irritated or anxious.** \| Strongly agree \| Agree \| Neutral \| Disagree \| Strongly disagree \| \| **5. You believe that improving the patient's quality of life is crucial for preventing stroke recurrence.** \| Strongly agree \| Agree \| Neutral \| Disagree \| Strongly disagree \| \| **6.** **You think that preventing stroke recurrence requires not only attention to physical health but also psychological counseling.** \| Strongly agree \| Agree \| Neutral \| Disagree \| Strongly disagree \| \| **7.** **You believe that preventing stroke recurrence is not necessary for recovery and that directly proceeding with acupuncture or other rehabilitation treatments is more effective.** \| Strongly agree \| Agree \| Neutral \| Disagree \| Strongly disagree \| \| **8. You think that quitting smoking and drinking and changing the patient’s previous lifestyle habits are difficult for both the patient and yourself.** \| Strongly agree \| Agree \| Neutral \| Disagree \| Strongly disagree \| \| **9. You feel that taking care of a stroke patient's daily needs is boring and physically exhausting.** \| Strongly agree \| Agree \| Neutral \| Disagree \| Strongly disagree \| \| **10.** **You believe the patient's confidence in recovery affects your caregiving attitude and enthusiasm.** \| Strongly agree \| Agree \| Neutral \| Disagree \| Strongly disagree \| | |
| --- | --- | --- | --- | --- | --- | --- | --- | --- | --- | --- | --- | --- | --- | --- | --- | --- | --- | --- | --- | --- | --- | --- | --- | --- | --- | --- | --- | --- | --- | --- | --- | --- | --- | --- | --- | --- | --- | --- | --- | --- | --- | --- | --- | --- | --- | --- | --- | --- | --- | --- | --- | --- | --- | --- | --- | --- | --- | --- | --- | --- | --- |
| **Part 4 Practices** |  |

| **1. You actively seek knowledge related to secondary stroke prevention and caregiving through various channels (e.g., books, internet, communication with doctors).** | Always | Often | Sometimes | Rarely | Never |
| --- | --- | --- | --- | --- | --- |
| **2. You guide the patient to engage in rehabilitation exercises, quit smoking and drinking, and maintain healthy lifestyle habits.** | Always | Often | Sometimes | Rarely | Never |
| **3. You provide psychological support and emotional counseling to help the patient manage negative emotions.** | Always | Often | Sometimes | Rarely | Never |
| **4. You assist the patient in maintaining personal and environmental hygiene (e.g., helping them change into clean clothes, maintain oral hygiene, and keep their rest area clean).** | Always | Often | Sometimes | Rarely | Never |
| **5. You provide nutritional support to the patient (e.g., preparing light and healthy meals).** | Always | Often | Sometimes | Rarely | Never |
| **6. You exchange experiences with other stroke caregivers regarding stroke recurrence prevention.** | Fully Compliant | Compliant | Neutral | Not Compliant | Very Not Compliant |
| **7. You accompany the patient to follow-up visits regularly and ensure adherence to medical advice while monitoring their recovery.** | Fully Compliant | Compliant | Neutral | Not Compliant | Very Not Compliant |
| **8. You pay attention to the patient’s cardiovascular and cerebrovascular health and support interventions such as endovascular procedures if needed.** | Fully Compliant | Compliant | Neutral | Not Compliant | Very Not Compliant |
| **9. You monitor the patient's communication with doctors and assist in enhancing their disease self-management skills.** | Fully Compliant | Compliant | Neutral | Not Compliant | Very Not Compliant |
| **10. You are consciously attentive to the patient’s needs and physical signs, monitoring indicators such as blood pressure and blood glucose, and provide comprehensive care.** | Fully Compliant | Compliant | Neutral | Not Compliant | Very Not Compliant |
