# Supplementary material for: Caregivers’ knowledge, attitudes, and practices regarding secondary prevention of stroke in patients with ischemic stroke
Source: Sci Rep. 2026 May 1;16:20209. doi: 10.1038/s41598-026-51436-8 (PMC13323690; doi:10.1038/s41598-026-51436-8)
Supplement: Supplementary file 2 — Supplementary Material 2 [file 41598_2026_51436_MOESM2_ESM.docx]

**Supplementary Table 1. Item–total correlations and Cronbach’s alpha if item deleted for the KAP questionnaire**

|  |  | Scale Mean if Item Deleted | Scale Variance if Item Deleted | Corrected Item-Total Correlation | Cronbach's Alpha if Item Deleted |
| --- | --- | --- | --- | --- | --- |
| Knowledge | K1 | 10.05 | 18.084 | 0.639 | 0.788 |
|  | K2 | 9.95 | 17.777 | 0.705 | 0.782 |
|  | K3 | 9.91 | 17.565 | 0.686 | 0.781 |
|  | K4 | 10.00 | 17.580 | 0.683 | 0.781 |
|  | K5 | 9.87 | 17.742 | 0.667 | 0.784 |
|  | K6 | 9.87 | 17.671 | 0.712 | 0.781 |
|  | K7 | 9.88 | 17.538 | 0.701 | 0.780 |
|  | K8 | 9.68 | 17.999 | 0.639 | 0.787 |
|  | K9 | 6.64 | 14.169 | 0.308 | 0.919 |
| Attitude | A1 | 30.54 | 20.651 | 0.281 | 0.675 |
|  | A2 | 30.77 | 21.081 | 0.220 | 0.683 |
|  | A3 | 31.40 | 17.604 | 0.383 | 0.659 |
|  | A4 | 31.45 | 17.456 | 0.530 | 0.627 |
|  | A5 | 30.68 | 21.128 | 0.228 | 0.682 |
|  | A6 | 30.63 | 20.886 | 0.263 | 0.678 |
|  | A7 | 31.39 | 17.683 | 0.469 | 0.639 |
|  | A8 | 31.71 | 18.217 | 0.358 | 0.663 |
|  | A9 | 31.52 | 17.643 | 0.489 | 0.635 |
|  | A10 | 31.97 | 19.938 | 0.228 | 0.687 |
| Practice | P1 | 35.63 | 31.126 | 0.583 | 0.894 |
|  | P2 | 35.01 | 30.885 | 0.693 | 0.884 |
|  | P3 | 35.14 | 30.248 | 0.750 | 0.879 |
|  | P4 | 34.88 | 31.310 | 0.744 | 0.880 |
|  | P5 | 34.91 | 31.448 | 0.696 | 0.883 |
|  | P6 | 34.96 | 33.031 | 0.613 | 0.889 |
|  | P7 | 34.56 | 34.170 | 0.634 | 0.889 |
|  | P8 | 34.78 | 33.922 | 0.542 | 0.893 |
|  | P9 | 34.59 | 34.184 | 0.633 | 0.889 |
|  | P10 | 34.51 | 34.049 | 0.638 | 0.888 |
| Total | K1 | 83.50 | 107.321 | 0.286 | 0.824 |
|  | K2 | 83.40 | 106.731 | 0.335 | 0.823 |
|  | K3 | 83.36 | 106.099 | 0.356 | 0.823 |
|  | K4 | 83.44 | 106.001 | 0.364 | 0.822 |
|  | K5 | 83.32 | 105.664 | 0.402 | 0.822 |
|  | K6 | 83.32 | 105.850 | 0.403 | 0.822 |
|  | K7 | 83.33 | 106.136 | 0.358 | 0.823 |
|  | K8 | 83.13 | 106.433 | 0.353 | 0.823 |
|  | K9 | 80.09 | 105.076 | 0.075 | 0.848 |
|  | A1 | 80.05 | 105.258 | 0.361 | 0.822 |
|  | A2 | 80.28 | 105.369 | 0.363 | 0.822 |
|  | A3 | 80.90 | 102.997 | 0.265 | 0.827 |
|  | A4 | 80.96 | 103.003 | 0.339 | 0.823 |
|  | A5 | 80.19 | 106.520 | 0.295 | 0.824 |
|  | A6 | 80.13 | 105.385 | 0.374 | 0.822 |
|  | A7 | 80.90 | 102.801 | 0.331 | 0.823 |
|  | A8 | 81.22 | 105.967 | 0.160 | 0.831 |
|  | A9 | 81.02 | 103.356 | 0.312 | 0.824 |
|  | A10 | 81.48 | 108.243 | 0.084 | 0.833 |
|  | P1 | 81.03 | 100.835 | 0.398 | 0.820 |
|  | P2 | 80.41 | 98.895 | 0.556 | 0.814 |
|  | P3 | 80.55 | 98.948 | 0.548 | 0.814 |
|  | P4 | 80.28 | 99.793 | 0.578 | 0.814 |
|  | P5 | 80.32 | 99.795 | 0.553 | 0.814 |
|  | P6 | 80.37 | 102.534 | 0.458 | 0.818 |
|  | P7 | 79.97 | 103.656 | 0.501 | 0.818 |
|  | P8 | 80.19 | 103.510 | 0.420 | 0.820 |
|  | P9 | 80.00 | 103.206 | 0.537 | 0.817 |
|  | P10 | 79.91 | 102.922 | 0.547 | 0.817 |

**Supplementary Table 2. Floor and ceiling effects of KAP domain scores**

|  | **Floor effect** | | **Ceiling effect** | |
| --- | --- | --- | --- | --- |
|  | **Value** | **N (Percent)** | **Value** | **N (Percent)** |
| Knowledge | 0 | 1 (0.2%) | 22 | 13 (2.1%) |
| Attitude | 24 | 1 (0.2%) | 50 | 4 (0.6%) |
| Practice | 19 | 2 (0.3%) | 50 | 66 (10.5%) |

**Supplementary Table 3. SEM fit indicators**

| **Model fit indicator** | **Ref.** | **Measured results** |
| --- | --- | --- |
| **CMIN/DF** | 1-3 excellent，3-5 good | 2.787 |
| **RMSEA** | <0.08 good | 0.055 |
| **IFI** | >0.8 good | 0.915 |
| **TLI** | >0.8 good | 0.904 |
| **CFI** | >0.8 good | 0.915 |
